# Dichlorido{*N*-[2-(diphenyl­phosphan­yl)benzyl­idene]-2-(thio­phen-2-yl)ethan­amine-κ^2^ *P*,*N*}platinum(II) dichloro­methane hemisolvate

**DOI:** 10.1107/S1600536811056108

**Published:** 2012-01-07

**Authors:** Haleden Chiririwa, Alfred Muller

**Affiliations:** a Research Center for Synthesis and Catalysis, Department of Chemistry, University of Johannesburg (APK Campus), PO Box 524, Auckland Park, Johannesburg 2006, South Africa

## Abstract

The crystal structure of the title compound, [PtCl_2_(C_25_H_22_NPS)]·0.5CH_2_Cl_2_, was determined to establish the coordination properties of the (phosphan­yl)benzyl­idene–methanamine ligand to platinum. In the unit cell two mol­ecules of *cis*-[PtCl_2_(C_25_H_22_NPS)] are accompanied by a dichloro­methane solvent mol­ecule. The square-planar Pt^2+^ coordination sphere is slightly distorted with the bidentate ligand coordinated *via* the P and the amine N atoms, and the Cl atoms located *cis* at the two remaining coordination sites. Parts of the thiophene ring and the solvate molecule were modeled as disordered with occupancy ratios of 0.55 (2):0.45 (2) and 0.302 (10):0.198 (10), respectively. Weak C—H⋯Cl inter­actions stabilize the crystal packing.

## Related literature

For background to related structures, see: Chiririwa *et al.* (2011 ▶); Chiririwa & Meijboom (2011*a* ▶,*b* ▶,*c* ▶); Ghilardi *et al.* (1992 ▶); Sanchez *et al.* (1998 ▶, 2001 ▶); Coleman *et al.* (2001 ▶). For Pt—N and Pt—P bond lengths in similar platinum(II) complexes, see: Ankersmit *et al.* (1996 ▶). For background to weak hydrogen-bonding inter­actions, see Steiner (1996 ▶). 
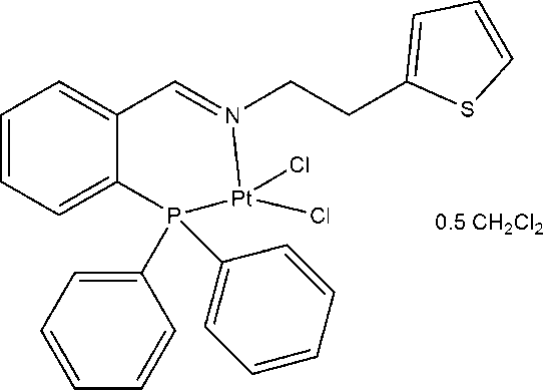


## Experimental

### 

#### Crystal data

[PtCl_2_(C_25_H_22_NPS)]·0.5CH_2_Cl_2_
*M* *_r_* = 707.92
Monoclinic, 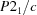

*a* = 9.9635 (7) Å
*b* = 19.0185 (14) Å
*c* = 16.0155 (9) Å
β = 122.751 (3)°
*V* = 2552.3 (3) Å^3^
*Z* = 4
Mo *K*α radiation
μ = 5.97 mm^−1^
*T* = 173 K
0.08 × 0.05 × 0.03 mm

#### Data collection

Bruker APEXII 4K-CCD diffractometer
Absorption correction: multi-scan (*SADABS*; Bruker, 2007 ▶) *T*_min_ = 0.647, *T*_max_ = 0.841
30158 measured reflections
6593 independent reflections
5165 reflections with *I* > 2σ(*I*)
*R*_int_ = 0.052

#### Refinement

*R*[*F* ^2^ > 2σ(*F* ^2^)] = 0.028
*wR*(*F* ^2^) = 0.058
*S* = 1.01
6593 reflections
333 parameters
146 restraints
H-atom parameters constrained
Δρ_max_ = 0.84 e Å^−3^
Δρ_min_ = −0.62 e Å^−3^

### 

Data collection: *APEX2* (Bruker, 2007 ▶); cell refinement: *SAINT* (Bruker, 2007 ▶); data reduction: *SAINT* and *XPREP* (Bruker, 2007 ▶); program(s) used to solve structure: *SIR97* (Altomare *et al.*, 1999 ▶); program(s) used to refine structure: *SHELXL97* (Sheldrick, 2008 ▶); molecular graphics: *DIAMOND* (Brandenburg & Putz, 2005 ▶); software used to prepare material for publication: *WinGX* (Farrugia, 1999 ▶).

## Supplementary Material

Crystal structure: contains datablock(s) global, I. DOI: 10.1107/S1600536811056108/nr2014sup1.cif

Structure factors: contains datablock(s) I. DOI: 10.1107/S1600536811056108/nr2014Isup2.hkl

Additional supplementary materials:  crystallographic information; 3D view; checkCIF report

## Figures and Tables

**Table 1:** Hydrogen-bond geometry (Å, °)

*D*—H⋯*A*	*D*—H	H⋯*A*	*D*⋯*A*	*D*—H⋯*A*
C7—H7⋯Cl2^i^	0.95	2.71	3.486 (4)	139
C12—H12⋯Cl1^ii^	0.95	2.71	3.639 (4)	168

Symmetry codes: (i) 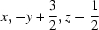; (ii) 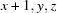.

**Fig. 1.**
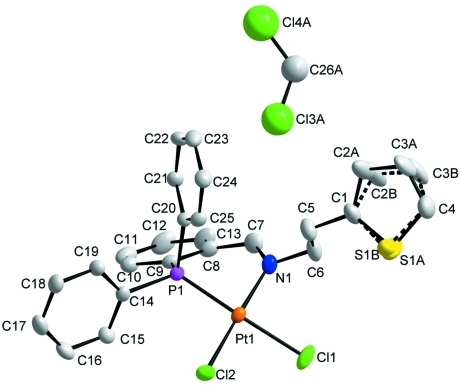
View of title compound showing displacement ellipsoids (drawn at a 30% probability level) and labeling. For clarity: a) hydrogen atoms omitted, b) bonds in part B of the disordered thiophene indicated with dotted lines, and c) minor component as well as symmetry generated disordered parts of dichloromethane created by inversion center omitted.

**Fig. 2.**
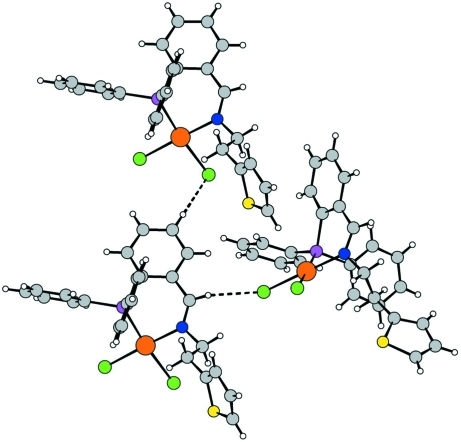
Partial packing diagram of title compound to illustrate the weak hydrogen bonding stabilizing crystal packing. All disordered components omitted for clarity.

## supplementary crystallographic information

### Comment

Platinum complexes with (phosphanyl)benzylidene-methanamine ligands have been used as catalysts or catalyst precursors for a variety of organic reactions. Our group and others have recently been interested in these types of complexes and have reported several of these types of complexes (Chiririwa *et al.*, 2011; Chiririwa & Meijboom, 2011*a*, 2011*b*, 2011*c*; Ghilardi *et al.*, 1992; Sanchez *et al.*, (1998, 2001) and Coleman *et al.*, 2001).

The title compound (see Fig. 1) crystallize with a dichloromethane solvate, disordered over an inversion center, for each pair of *cis*-[PtCl_2_(C_25_H_21_NSP)] molecules. The square planar Pt coordination sphere is slightly distorted (Pt1 displaced -0.0275 (7)) Å from the plane formed by Pt1, P1, N1, Cl1 and Cl2 respectively; r.m.s. deviation of fitted atoms = 0.0423 Å). The distortion is featured most prominently in the N1—Pt1—P1 angle of 86.17 (10)° *versus*. Cl1—Pt1—Cl2 = 89.08 (4)°, indicating some ring strain induced by the chelation of the bidentate ligand. The average Pt—N and Pt—P bond lengths of 2.030 (3) and 2.2089 (9) Å, respectively are in the range expected for similar platinum(II) complexes (Ankersmit *et al.*(1996)). The initial refinement model of the compound showed some large displacement parameters for the thiophene moiety as well as the dichloromethane solvent. These disorders were elucidated to give an improved model (details can be found under the experimental refinement section). Two weak C—H···Cl interactions (Steiner, 1996) aid in the stabilization of the crystal structure (see Table 1, Fig. 2).

### Experimental

To a dry CH_2_Cl_2_ (10 ml) solution of the precursor [Pt(COD)Cl_2_] was added an equimolar amount of (2-(diphenylphosphanyl) benzylidene)(thiophen-2-yl)methanamine in CH_2_Cl_2_ (10 ml), and stirred at room temperature for 2 hrs. The solvent was reduced and the complex precipitated out on addition of hexane, filtered off, washed with Et_2_O (2×5 ml) and dried under vacuum for 4 hrs affording a yellow precipitate in 72% yield. Crystals suitable for X-ray structure determination were obtained by recrystallization form a CH_2_Cl_2_-hexane mixture at room temperature.

### Refinement

All hydrogen atoms were positioned in geometrically idealized positions with C—H = 0.99 Å and 0.95 Å for methylene and aromatic H atoms respectively. All hydrogen atoms were allowed to ride on their parent atoms with *U*_iso_(H) = 1.2*U*_eq_. A disorder refinement model was applied to the thiophene that showed large displacements at C2, C3 and S1. Geometrical (FLAT) restraints were applied to keep the rings C1, C2A/B, C3A/B, C4, S1A/B planar. Ellipsoid displacement (SIMU and DELU) restraints were also applied to the disordered moiety. The occupation parameters of the two disordered tiophene fragments were linked to a free variable so that the two sites add to unity. This showed a distribution of 0.54903:0.45097., The dichloromethane solvate was also refined as disordered on two positions in the asymmetric unit. This resulted in four disordered positions for each dichloromethane at each solvent accessible site in the crystal lattice. The occupancies of these sites were linked to a free variable to add to unity and refined to a ratio of 0.60331:0.39669 (based on the asymmetric unit fraction). To keep refinement stable geometrical (DIFX and DANG) restraints were applied to the C—Cl bonds and Cl···Cl distances All the above restraints were applied with the default standard deviations. The atoms of the solvate molecule was left isotropic due to the extensive nature of the disorder. The highest residual electron density of 0.84 e.Å^-3^ is 0.89 Å from Pt1 representing no physical meaning.

### Crystal data

**Table:**

[PtCl_2_(C_25_H_22_NPS)]·0.5CH_2_Cl_2_	*F*(000) = 1372
*M**_r_* = 707.92	*D*_x_ = 1.842 Mg m^−^^3^
Monoclinic, *P*2_1_/*c*	Mo *K*α radiation, λ = 0.71073 Å
Hall symbol: -P 2ybc	Cell parameters from 5962 reflections
*a* = 9.9635 (7) Å	θ = 2.6–28.2°
*b* = 19.0185 (14) Å	µ = 5.97 mm^−^^1^
*c* = 16.0155 (9) Å	*T* = 173 K
β = 122.751 (3)°	Block, yellow
*V* = 2552.3 (3) Å^3^	0.08 × 0.05 × 0.03 mm
*Z* = 4	

### Data collection

**Table:**

Bruker APEXII 4K-CCD diffractometer	6593 independent reflections
graphite	5165 reflections with *I* > 2σ(*I*)
Detector resolution: 8.4 pixels mm^-1^	*R*_int_ = 0.052
φ and ω scans	θ_max_ = 28.7°, θ_min_ = 2.1°
Absorption correction: multi-scan (*SADABS*; Bruker, 2007)	*h* = −13→13
*T*_min_ = 0.647, *T*_max_ = 0.841	*k* = −25→25
30158 measured reflections	*l* = −21→21

### Refinement

**Table:**

Refinement on *F*^2^	Primary atom site location: structure-invariant direct methods
Least-squares matrix: full	Secondary atom site location: difference Fourier map
*R*[*F*^2^ > 2σ(*F*^2^)] = 0.028	Hydrogen site location: inferred from neighbouring sites
*wR*(*F*^2^) = 0.058	H-atom parameters constrained
*S* = 1.01	*w* = 1/[σ^2^(*F*_o_^2^) + (0.0216*P*)^2^ + 0.6014*P*] where *P* = (*F*_o_^2^ + 2*F*_c_^2^)/3
6593 reflections	(Δ/σ)_max_ = 0.002
333 parameters	Δρ_max_ = 0.84 e Å^−^^3^
146 restraints	Δρ_min_ = −0.62 e Å^−^^3^

### Special details

**Table:**

Experimental. The intensity data was collected on a Bruker Apex-II 4 K CCD diffractometer using an exposure time of 80 s/frame. A total of 1315 frames were collected with a frame width of 0.5° covering up to θ = 28.72° with 99.8% completeness accomplished.
Geometry. All e.s.d.'s (except the e.s.d. in the dihedral angle between two l.s. planes) are estimated using the full covariance matrix. The cell e.s.d.'s are taken into account individually in the estimation of e.s.d.'s in distances, angles and torsion angles; correlations between e.s.d.'s in cell parameters are only used when they are defined by crystal symmetry. An approximate (isotropic) treatment of cell e.s.d.'s is used for estimating e.s.d.'s involving l.s. planes.
Refinement. Refinement of *F*^2^ against ALL reflections. The weighted *R*-factor *wR* and goodness of fit *S* are based on *F*^2^, conventional *R*-factors *R* are based on *F*, with *F* set to zero for negative *F*^2^. The threshold expression of *F*^2^ > σ(*F*^2^) is used only for calculating *R*-factors(gt) *etc*. and is not relevant to the choice of reflections for refinement. *R*-factors based on *F*^2^ are statistically about twice as large as those based on *F*, and *R*- factors based on ALL data will be even larger.

### Fractional atomic coordinates and isotropic or equivalent isotropic displacement parameters (Å^2^)

**Table:**

	*x*	*y*	*z*	*U*_iso_*/*U*_eq_	Occ. (<1)
Pt1	0.557569 (15)	0.701063 (7)	0.420321 (11)	0.02063 (4)	
Cl1	0.38212 (12)	0.79833 (5)	0.36520 (9)	0.0382 (2)	
Cl2	0.57723 (11)	0.70634 (5)	0.56946 (7)	0.0318 (2)	
P1	0.73311 (10)	0.61536 (5)	0.46437 (7)	0.01889 (18)	
N1	0.5342 (4)	0.69787 (16)	0.2864 (2)	0.0278 (7)	
C1	0.1129 (5)	0.6300 (2)	0.1049 (3)	0.0385 (10)	
C2A	0.0513 (10)	0.5796 (8)	0.0305 (9)	0.056 (4)	0.55 (2)
H2A	0.1137	0.5437	0.0264	0.067*	0.55 (2)
C3A	−0.1149 (10)	0.5875 (9)	−0.0387 (9)	0.056 (4)	0.55 (2)
H3A	−0.1758	0.5578	−0.0947	0.067*	0.55 (2)
S1A	−0.0359 (10)	0.6794 (6)	0.0932 (7)	0.060 (2)	0.55 (2)
C2B	0.0654 (13)	0.6113 (12)	0.0100 (8)	0.069 (5)	0.45 (2)
H2B	0.136	0.5934	−0.0076	0.083*	0.45 (2)
C3B	−0.0994 (13)	0.6213 (13)	−0.0587 (8)	0.060 (5)	0.45 (2)
H3B	−0.1501	0.6136	−0.1281	0.072*	0.45 (2)
S1B	−0.0452 (11)	0.6648 (7)	0.1052 (7)	0.0434 (19)	0.45 (2)
C4	−0.1785 (5)	0.6432 (3)	−0.0158 (3)	0.0511 (12)	
H4A	−0.286	0.6588	−0.0553	0.061*	0.45 (2)
H4B	−0.2912	0.6461	−0.0484	0.061*	0.55 (2)
C5	0.2814 (5)	0.6328 (2)	0.1939 (3)	0.0382 (10)	
H5A	0.3404	0.5912	0.1932	0.046*	
H5B	0.2795	0.6306	0.255	0.046*	
C6	0.3689 (5)	0.6990 (2)	0.1964 (3)	0.0369 (10)	
H6A	0.3728	0.7015	0.136	0.044*	
H6B	0.3115	0.741	0.1979	0.044*	
C7	0.6488 (5)	0.6932 (2)	0.2717 (3)	0.0318 (9)	
H7	0.6203	0.6955	0.2047	0.038*	
C8	0.8184 (5)	0.68468 (19)	0.3477 (3)	0.0272 (8)	
C9	0.8769 (4)	0.64993 (18)	0.4383 (3)	0.0229 (8)	
C10	1.0409 (4)	0.64245 (19)	0.5044 (3)	0.0267 (8)	
H10	1.0809	0.6181	0.5652	0.032*	
C11	1.1464 (5)	0.6705 (2)	0.4817 (3)	0.0345 (10)	
H11	1.2581	0.6662	0.528	0.041*	
C12	1.0910 (5)	0.7042 (2)	0.3935 (4)	0.0422 (11)	
H12	1.1639	0.723	0.3784	0.051*	
C13	0.9267 (5)	0.7108 (2)	0.3254 (3)	0.0365 (10)	
H13	0.8882	0.7332	0.2635	0.044*	
C14	0.8472 (4)	0.58128 (18)	0.5904 (3)	0.0209 (7)	
C15	0.9314 (4)	0.6271 (2)	0.6700 (3)	0.0273 (8)	
H15	0.9295	0.6762	0.6586	0.033*	
C16	1.0187 (5)	0.6010 (2)	0.7665 (3)	0.0341 (9)	
H16	1.0763	0.6323	0.8208	0.041*	
C17	1.0217 (5)	0.5291 (2)	0.7835 (3)	0.0331 (9)	
H17	1.0799	0.5114	0.8495	0.04*	
C18	0.9395 (5)	0.4836 (2)	0.7041 (3)	0.0302 (9)	
H18	0.943	0.4344	0.7156	0.036*	
C19	0.8526 (4)	0.50892 (19)	0.6080 (3)	0.0246 (8)	
H19	0.7964	0.4773	0.5538	0.029*	
C20	0.6463 (4)	0.53857 (17)	0.3865 (3)	0.0201 (7)	
C21	0.7114 (4)	0.50677 (19)	0.3381 (3)	0.0255 (8)	
H21	0.8031	0.5265	0.3432	0.031*	
C22	0.6429 (5)	0.4463 (2)	0.2825 (3)	0.0306 (9)	
H22	0.6885	0.4246	0.25	0.037*	
C23	0.5093 (5)	0.4177 (2)	0.2741 (3)	0.0318 (9)	
H23	0.463	0.3762	0.2361	0.038*	
C24	0.4419 (4)	0.44923 (19)	0.3211 (3)	0.0288 (9)	
H24	0.3494	0.4295	0.315	0.035*	
C25	0.5098 (4)	0.50953 (18)	0.3769 (3)	0.0234 (8)	
H25	0.4633	0.5313	0.4088	0.028*	
C26A	0.485 (3)	0.5266 (10)	−0.0366 (13)	0.070 (6)*	0.302 (10)
H26A	0.5187	0.5656	−0.0625	0.084*	0.302 (10)
H26B	0.3693	0.5203	−0.0843	0.084*	0.302 (10)
Cl3A	0.5086 (19)	0.5549 (5)	0.0677 (8)	0.099 (3)*	0.302 (10)
Cl4A	0.5746 (14)	0.4531 (5)	−0.0434 (8)	0.121 (4)*	0.302 (10)
C26B	0.564 (3)	0.5398 (9)	−0.0142 (17)	0.047 (7)*	0.198 (10)
H26C	0.6572	0.5502	−0.0185	0.057*	0.198 (10)
H26D	0.4676	0.5612	−0.072	0.057*	0.198 (10)
Cl3B	0.591 (3)	0.5697 (8)	0.0906 (11)	0.122 (5)*	0.198 (10)
Cl4B	0.5390 (15)	0.4497 (6)	−0.0105 (12)	0.090 (4)*	0.198 (10)

### Atomic displacement parameters (Å^2^)

**Table:**

	*U*^11^	*U*^22^	*U*^33^	*U*^12^	*U*^13^	*U*^23^
Pt1	0.01969 (7)	0.01735 (7)	0.02733 (8)	0.00077 (6)	0.01435 (6)	0.00296 (7)
Cl1	0.0329 (5)	0.0267 (5)	0.0624 (7)	0.0114 (4)	0.0307 (5)	0.0171 (5)
Cl2	0.0343 (5)	0.0353 (5)	0.0322 (5)	0.0034 (4)	0.0221 (4)	−0.0041 (4)
P1	0.0200 (4)	0.0179 (4)	0.0209 (5)	−0.0001 (3)	0.0125 (4)	0.0013 (4)
N1	0.0254 (16)	0.0288 (17)	0.0290 (18)	0.0021 (14)	0.0145 (14)	0.0078 (15)
C1	0.030 (2)	0.057 (3)	0.022 (2)	0.0020 (19)	0.0097 (17)	0.0003 (19)
C2A	0.026 (4)	0.097 (9)	0.036 (6)	0.011 (5)	0.011 (4)	−0.024 (6)
C3A	0.027 (4)	0.096 (9)	0.029 (6)	−0.004 (5)	0.005 (4)	−0.022 (6)
S1A	0.048 (2)	0.036 (3)	0.068 (4)	0.0109 (17)	0.012 (2)	−0.009 (2)
C2B	0.033 (5)	0.141 (14)	0.029 (6)	0.006 (8)	0.014 (4)	−0.018 (8)
C3B	0.038 (6)	0.113 (13)	0.020 (5)	−0.002 (7)	0.009 (4)	−0.006 (7)
S1B	0.042 (2)	0.051 (5)	0.033 (2)	0.013 (2)	0.0175 (18)	−0.005 (2)
C4	0.033 (2)	0.059 (3)	0.040 (3)	0.009 (2)	0.006 (2)	−0.002 (2)
C5	0.030 (2)	0.041 (2)	0.028 (2)	−0.0028 (18)	0.0059 (18)	0.0048 (19)
C6	0.030 (2)	0.046 (3)	0.026 (2)	0.0025 (19)	0.0096 (18)	0.013 (2)
C7	0.039 (2)	0.030 (2)	0.032 (2)	−0.0017 (18)	0.0224 (19)	0.0090 (18)
C8	0.032 (2)	0.024 (2)	0.036 (2)	−0.0011 (15)	0.0254 (19)	0.0005 (16)
C9	0.0273 (19)	0.0202 (18)	0.028 (2)	−0.0018 (15)	0.0195 (17)	−0.0026 (16)
C10	0.0253 (19)	0.027 (2)	0.030 (2)	−0.0024 (15)	0.0171 (18)	−0.0036 (17)
C11	0.026 (2)	0.031 (2)	0.051 (3)	−0.0060 (17)	0.024 (2)	−0.010 (2)
C12	0.041 (2)	0.035 (2)	0.071 (3)	−0.004 (2)	0.044 (3)	0.003 (2)
C13	0.045 (3)	0.031 (2)	0.051 (3)	0.0018 (18)	0.038 (2)	0.012 (2)
C14	0.0195 (17)	0.0241 (18)	0.0217 (19)	0.0019 (14)	0.0129 (15)	0.0023 (15)
C15	0.0240 (19)	0.027 (2)	0.026 (2)	−0.0034 (15)	0.0108 (17)	−0.0019 (16)
C16	0.029 (2)	0.041 (2)	0.022 (2)	−0.0067 (18)	0.0081 (17)	−0.0068 (19)
C17	0.029 (2)	0.046 (3)	0.022 (2)	0.0037 (18)	0.0123 (18)	0.0061 (19)
C18	0.033 (2)	0.027 (2)	0.029 (2)	0.0060 (17)	0.0162 (18)	0.0084 (17)
C19	0.029 (2)	0.0231 (19)	0.023 (2)	0.0015 (15)	0.0149 (17)	0.0030 (15)
C20	0.0224 (17)	0.0174 (17)	0.0195 (18)	−0.0007 (14)	0.0107 (15)	0.0001 (14)
C21	0.0245 (19)	0.029 (2)	0.025 (2)	0.0007 (16)	0.0151 (17)	−0.0014 (16)
C22	0.034 (2)	0.033 (2)	0.024 (2)	0.0030 (17)	0.0154 (18)	−0.0066 (17)
C23	0.031 (2)	0.0207 (19)	0.031 (2)	−0.0014 (16)	0.0092 (18)	−0.0039 (17)
C24	0.026 (2)	0.025 (2)	0.031 (2)	−0.0041 (16)	0.0123 (17)	0.0000 (17)
C25	0.0268 (19)	0.0220 (18)	0.027 (2)	0.0008 (15)	0.0179 (16)	0.0008 (16)

### Geometric parameters (Å, °)

**Table:**

Pt1—N1	2.029 (3)	C10—H10	0.95
Pt1—P1	2.2087 (9)	C11—C12	1.367 (6)
Pt1—Cl2	2.2907 (10)	C11—H11	0.95
Pt1—Cl1	2.3638 (9)	C12—C13	1.397 (6)
P1—C20	1.808 (4)	C12—H12	0.95
P1—C14	1.818 (4)	C13—H13	0.95
P1—C9	1.818 (4)	C14—C15	1.390 (5)
N1—C7	1.288 (5)	C14—C19	1.400 (5)
N1—C6	1.490 (5)	C15—C16	1.391 (5)
C1—C2B	1.373 (11)	C15—H15	0.95
C1—C2A	1.388 (10)	C16—C17	1.391 (6)
C1—C5	1.504 (5)	C16—H16	0.95
C1—S1A	1.678 (9)	C17—C18	1.383 (5)
C1—S1B	1.711 (9)	C17—H17	0.95
C2A—C3A	1.416 (9)	C18—C19	1.381 (5)
C2A—H2A	0.95	C18—H18	0.95
C3A—C4	1.382 (10)	C19—H19	0.95
C3A—H3A	0.95	C20—C21	1.389 (5)
S1A—C4	1.690 (9)	C20—C25	1.397 (5)
C2B—C3B	1.410 (11)	C21—C22	1.387 (5)
C2B—H2B	0.95	C21—H21	0.95
C3B—C4	1.362 (11)	C22—C23	1.376 (5)
C3B—H3B	0.95	C22—H22	0.95
S1B—C4	1.704 (10)	C23—C24	1.386 (5)
C4—H4A	0.95	C23—H23	0.95
C4—H4B	0.95	C24—C25	1.385 (5)
C5—C6	1.519 (5)	C24—H24	0.95
C5—H5A	0.99	C25—H25	0.95
C5—H5B	0.99	C26A—Cl3A	1.647 (16)
C6—H6A	0.99	C26A—Cl4A	1.692 (15)
C6—H6B	0.99	C26A—H26A	0.99
C7—C8	1.462 (6)	C26A—H26B	0.99
C7—H7	0.95	C26B—Cl3B	1.648 (16)
C8—C13	1.397 (5)	C26B—Cl4B	1.737 (16)
C8—C9	1.403 (5)	C26B—H26C	0.99
C9—C10	1.393 (5)	C26B—H26D	0.99
C10—C11	1.391 (5)		
			
N1—Pt1—P1	86.20 (9)	C13—C8—C7	116.9 (4)
N1—Pt1—Cl2	178.38 (9)	C9—C8—C7	124.0 (3)
P1—Pt1—Cl2	95.34 (3)	C10—C9—C8	119.6 (3)
N1—Pt1—Cl1	89.42 (9)	C10—C9—P1	122.3 (3)
P1—Pt1—Cl1	174.06 (3)	C8—C9—P1	118.1 (3)
Cl2—Pt1—Cl1	89.08 (4)	C11—C10—C9	120.3 (4)
C20—P1—C14	104.86 (16)	C11—C10—H10	119.9
C20—P1—C9	106.01 (16)	C9—C10—H10	119.9
C14—P1—C9	106.69 (16)	C12—C11—C10	120.7 (4)
C20—P1—Pt1	112.03 (12)	C12—C11—H11	119.7
C14—P1—Pt1	121.94 (11)	C10—C11—H11	119.7
C9—P1—Pt1	104.23 (12)	C11—C12—C13	119.7 (4)
C7—N1—C6	116.7 (3)	C11—C12—H12	120.1
C7—N1—Pt1	126.1 (3)	C13—C12—H12	120.1
C6—N1—Pt1	117.2 (2)	C8—C13—C12	120.6 (4)
C2B—C1—C5	126.7 (6)	C8—C13—H13	119.7
C2A—C1—C5	125.9 (5)	C12—C13—H13	119.7
C2B—C1—S1A	105.5 (6)	C15—C14—C19	119.6 (3)
C2A—C1—S1A	109.7 (5)	C15—C14—P1	120.0 (3)
C5—C1—S1A	123.5 (4)	C19—C14—P1	120.4 (3)
C2B—C1—S1B	109.7 (6)	C14—C15—C16	119.9 (4)
C2A—C1—S1B	106.8 (5)	C14—C15—H15	120
C5—C1—S1B	122.8 (4)	C16—C15—H15	120
C1—C2A—C3A	112.6 (7)	C17—C16—C15	120.2 (4)
C1—C2A—H2A	123.7	C17—C16—H16	119.9
C3A—C2A—H2A	123.7	C15—C16—H16	119.9
C4—C3A—C2A	112.6 (7)	C18—C17—C16	119.7 (4)
C4—C3A—H3A	123.7	C18—C17—H17	120.1
C2A—C3A—H3A	123.7	C16—C17—H17	120.1
C1—S1A—C4	94.9 (5)	C19—C18—C17	120.5 (4)
C1—C2B—C3B	112.8 (8)	C19—C18—H18	119.7
C1—C2B—H2B	123.6	C17—C18—H18	119.7
C3B—C2B—H2B	123.6	C18—C19—C14	120.0 (4)
C4—C3B—C2B	113.3 (8)	C18—C19—H19	120
C4—C3B—H3B	123.4	C14—C19—H19	120
C2B—C3B—H3B	123.4	C21—C20—C25	119.1 (3)
C4—S1B—C1	93.2 (4)	C21—C20—P1	122.9 (3)
C3B—C4—S1A	104.7 (6)	C25—C20—P1	118.0 (3)
C3A—C4—S1A	109.6 (5)	C22—C21—C20	120.2 (3)
C3B—C4—S1B	109.9 (6)	C22—C21—H21	119.9
C3A—C4—S1B	107.4 (5)	C20—C21—H21	119.9
C3B—C4—H4A	120.7	C23—C22—C21	120.3 (4)
C3A—C4—H4A	125.2	C23—C22—H22	119.9
S1A—C4—H4A	125.2	C21—C22—H22	119.9
S1B—C4—H4A	126	C22—C23—C24	120.2 (4)
C3B—C4—H4B	125.1	C22—C23—H23	119.9
C3A—C4—H4B	118.3	C24—C23—H23	119.9
S1A—C4—H4B	128.9	C25—C24—C23	119.8 (4)
S1B—C4—H4B	125	C25—C24—H24	120.1
C1—C5—C6	112.6 (3)	C23—C24—H24	120.1
C1—C5—H5A	109.1	C24—C25—C20	120.4 (3)
C6—C5—H5A	109.1	C24—C25—H25	119.8
C1—C5—H5B	109.1	C20—C25—H25	119.8
C6—C5—H5B	109.1	Cl3A—C26A—Cl4A	123.0 (11)
H5A—C5—H5B	107.8	Cl3A—C26A—H26A	106.6
N1—C6—C5	109.4 (3)	Cl4A—C26A—H26A	106.6
N1—C6—H6A	109.8	Cl3A—C26A—H26B	106.6
C5—C6—H6A	109.8	Cl4A—C26A—H26B	106.6
N1—C6—H6B	109.8	H26A—C26A—H26B	106.6
C5—C6—H6B	109.8	Cl3B—C26B—Cl4B	104.7 (12)
H6A—C6—H6B	108.2	Cl3B—C26B—H26C	110.8
N1—C7—C8	126.5 (4)	Cl4B—C26B—H26C	110.8
N1—C7—H7	116.7	Cl3B—C26B—H26D	110.8
C8—C7—H7	116.7	Cl4B—C26B—H26D	110.8
C13—C8—C9	119.1 (4)	H26C—C26B—H26D	108.9
			
N1—Pt1—P1—C20	−59.67 (15)	Pt1—N1—C7—C8	4.2 (6)
Cl2—Pt1—P1—C20	119.82 (12)	N1—C7—C8—C13	−152.5 (4)
N1—Pt1—P1—C14	174.99 (16)	N1—C7—C8—C9	30.0 (6)
Cl2—Pt1—P1—C14	−5.52 (14)	C13—C8—C9—C10	0.3 (5)
N1—Pt1—P1—C9	54.51 (15)	C7—C8—C9—C10	177.7 (4)
Cl2—Pt1—P1—C9	−126.00 (13)	C13—C8—C9—P1	−179.1 (3)
P1—Pt1—N1—C7	−43.9 (3)	C7—C8—C9—P1	−1.7 (5)
Cl1—Pt1—N1—C7	132.1 (3)	C20—P1—C9—C10	−105.8 (3)
P1—Pt1—N1—C6	134.3 (3)	C14—P1—C9—C10	5.6 (3)
Cl1—Pt1—N1—C6	−49.8 (3)	Pt1—P1—C9—C10	135.8 (3)
C2B—C1—C2A—C3A	−82.1 (14)	C20—P1—C9—C8	73.6 (3)
C5—C1—C2A—C3A	174.8 (7)	C14—P1—C9—C8	−175.0 (3)
S1A—C1—C2A—C3A	5.3 (10)	Pt1—P1—C9—C8	−44.7 (3)
S1B—C1—C2A—C3A	18.3 (10)	C8—C9—C10—C11	1.3 (5)
C1—C2A—C3A—C4	−0.7 (12)	P1—C9—C10—C11	−179.3 (3)
C2B—C1—S1A—C4	25.5 (9)	C9—C10—C11—C12	−1.6 (6)
C2A—C1—S1A—C4	−6.6 (7)	C10—C11—C12—C13	0.2 (6)
C5—C1—S1A—C4	−176.4 (4)	C9—C8—C13—C12	−1.8 (6)
S1B—C1—S1A—C4	−86 (3)	C7—C8—C13—C12	−179.3 (4)
C2A—C1—C2B—C3B	86.2 (15)	C11—C12—C13—C8	1.5 (6)
C5—C1—C2B—C3B	−173.5 (9)	C20—P1—C14—C15	179.5 (3)
S1A—C1—C2B—C3B	−16.4 (13)	C9—P1—C14—C15	67.3 (3)
S1B—C1—C2B—C3B	−3.8 (12)	Pt1—P1—C14—C15	−52.0 (3)
C1—C2B—C3B—C4	−3.9 (16)	C20—P1—C14—C19	0.4 (3)
C2B—C1—S1B—C4	8.0 (9)	C9—P1—C14—C19	−111.8 (3)
C2A—C1—S1B—C4	−24.5 (8)	Pt1—P1—C14—C19	128.9 (2)
C5—C1—S1B—C4	178.2 (4)	C19—C14—C15—C16	−0.9 (5)
S1A—C1—S1B—C4	81 (2)	P1—C14—C15—C16	180.0 (3)
C2B—C3B—C4—C3A	−81.7 (14)	C14—C15—C16—C17	−0.1 (6)
C2B—C3B—C4—S1A	21.9 (13)	C15—C16—C17—C18	1.0 (6)
C2B—C3B—C4—S1B	9.7 (15)	C16—C17—C18—C19	−1.0 (6)
C2A—C3A—C4—C3B	82.5 (13)	C17—C18—C19—C14	0.1 (6)
C2A—C3A—C4—S1A	−4.1 (11)	C15—C14—C19—C18	0.9 (5)
C2A—C3A—C4—S1B	−17.4 (11)	P1—C14—C19—C18	−180.0 (3)
C1—S1A—C4—C3B	−27.5 (9)	C14—P1—C20—C21	−98.8 (3)
C1—S1A—C4—C3A	6.2 (8)	C9—P1—C20—C21	13.8 (4)
C1—S1A—C4—S1B	88 (2)	Pt1—P1—C20—C21	126.9 (3)
C1—S1B—C4—C3B	−10.1 (10)	C14—P1—C20—C25	79.7 (3)
C1—S1B—C4—C3A	24.2 (8)	C9—P1—C20—C25	−167.6 (3)
C1—S1B—C4—S1A	−78 (2)	Pt1—P1—C20—C25	−54.5 (3)
C2B—C1—C5—C6	79.4 (13)	C25—C20—C21—C22	−1.1 (5)
C2A—C1—C5—C6	118.0 (10)	P1—C20—C21—C22	177.5 (3)
S1A—C1—C5—C6	−73.9 (8)	C20—C21—C22—C23	0.5 (6)
S1B—C1—C5—C6	−89.1 (7)	C21—C22—C23—C24	0.2 (6)
C7—N1—C6—C5	113.9 (4)	C22—C23—C24—C25	−0.3 (6)
Pt1—N1—C6—C5	−64.4 (4)	C23—C24—C25—C20	−0.3 (6)
C1—C5—C6—N1	179.9 (4)	C21—C20—C25—C24	1.0 (5)
C6—N1—C7—C8	−174.0 (4)	P1—C20—C25—C24	−177.6 (3)

### Hydrogen-bond geometry (Å, °)

**Table:**

*D*—H···*A*	*D*—H	H···*A*	*D*···*A*	*D*—H···*A*
C7—H7···Cl2^i^	0.95	2.71	3.486 (4)	139
C12—H12···Cl1^ii^	0.95	2.71	3.639 (4)	168.

Symmetry codes: (i) *x*, −*y*+3/2, *z*−1/2; (ii) *x*+1, *y*, *z*.
